# EEG Measures of Value-Directed Strategic Processing in Older Adults With and Without Mild Cognitive Impairment

**DOI:** 10.1093/geroni/igaa057.937

**Published:** 2020-12-16

**Authors:** Lydia Nguyen, Shraddha Shende, Daniel Llano, Raksha Mudar

**Affiliations:** 1 University of Illinois at Urbana-Champaign, Champaign, Illinois, United States; 2 University of Illinois at Urbana-Champaign, Urbana, Illinois, United States

## Abstract

Value-directed strategic processing is important for daily functioning. It allows selective processing of important information and inhibition of irrelevant information. This ability is relatively preserved in normal cognitive aging, but it is unclear if mild cognitive impairment (MCI) affects strategic processing and its underlying neurophysiological mechanisms. The current study examined behavioral and EEG spectral power differences between 16 cognitively normal older adults (CNOA; mean age: 74.5 ± 4.0 years) and 16 individuals with MCI (mean age: 77.1 ± 4.3 years) linked to a value-directed strategic processing task. The task used five unique word lists where words were assigned high- or low-value based on letter case and were presented sequentially while EEG was recorded. Participants were instructed to recall as many words as possible after each list to maximize their score. Results revealed no group differences in recall of low-value words, but individuals with MCI recalled significantly fewer high-value words and total number of words relative to CNOA. Group differences were observed in theta and alpha bands for low-value words, with greater synchronized theta power for CNOA than MCI and greater desynchronized alpha power for MCI than CNOA. Collectively, these findings demonstrate that more effortful neural processing of low-value words in the MCI group, relative to the CNOA group, allowed them to match their behavioral performance to the CNOA group. Individuals with MCI appear to utilize more cognitive resources to inhibit low-value information and might show memory-related benefits if taught strategies to focus on high-value information processing.

